# Research progress on HIV-1 structural proteins and antiviral therapies

**DOI:** 10.3389/fimmu.2026.1805597

**Published:** 2026-06-03

**Authors:** Huihan Wang, Jinsong Yuan, Hong Wang

**Affiliations:** Institute of Virology and AIDS Research, Center of Infectious Diseases and Pathogen Biology, Key Laboratory of Organ Regeneration and Transplantation of the Ministry of Education, The First Hospital of Jilin University, Changchun, China

**Keywords:** aids, antiviral therapies, HIV-1, inhibitors, structural proteins

## Abstract

Human Immunodeficiency Virus (HIV), including HIV-1 and HIV-2, is a retrovirus that causes Acquired Immunodeficiency Syndrome (AIDS). HIV primarily targets the human immune system, especially CD4^+^ T lymphocytes, leading to progressive immune system dysfunction and rendering patients susceptible to severe opportunistic infections and malignancies. HIV-1 accounts for the majority of global HIV infections, and most structural, mechanistic, and therapeutic studies have been conducted in HIV-1 systems. The structural proteins Gag, Pol, and Env of HIV-1 play a pivotal role in the viral life cycle, making their functional mechanisms and inhibitor development key to advancing anti-AIDS drug research. This article provides a narrative review of recent advances in antiviral drug development targeting these proteins and discusses future design strategies. It offers critical insights for accelerating next-generation anti-HIV drug development and advancing global AIDS control efforts.

## 1 Introduction

Human immunodeficiency virus (HIV), a retrovirus that includes HIV-1 and HIV-2, relies on the coordinated activity of various virally encoded proteins to complete its replication cycle. These components are essential not only for virion assembly and release but also play critical roles in mediating virus–host interactions (1). Because HIV-1 accounts for the majority of global HIV infections and most structural, mechanistic, and therapeutic studies have been conducted in HIV-1 systems, this review mainly focuses on HIV-1. The HIV-1 genome encodes nine genes, which include structural proteins (Gag, Pol, Env), accessory proteins (Vif, Vpu, Vpr) and regulatory proteins (Rev, Tat, Nef) (2). Among these, the structural proteins Gag, Pol and Env are particularly crucial. Gag and Pol are responsible for the assembly of new virions and the processing of viral proteins, while Env facilitates the virus’s entry into host cells by interacting with the host cell receptors. These proteins are not only indispensable for the virus’s replication cycle but also present attractive targets for antiviral interventions, as disrupting their functions can prevent viral assembly, maturation, and entry. This article is intended as a narrative review focusing on the structure and function of HIV-1 structural proteins, current antiviral drug development targeting these proteins, and potential directions for future drug design.

## 2 Structural and functional roles of HIV-1 structural proteins

### 2.1 Gag protein

**Figure 1:**
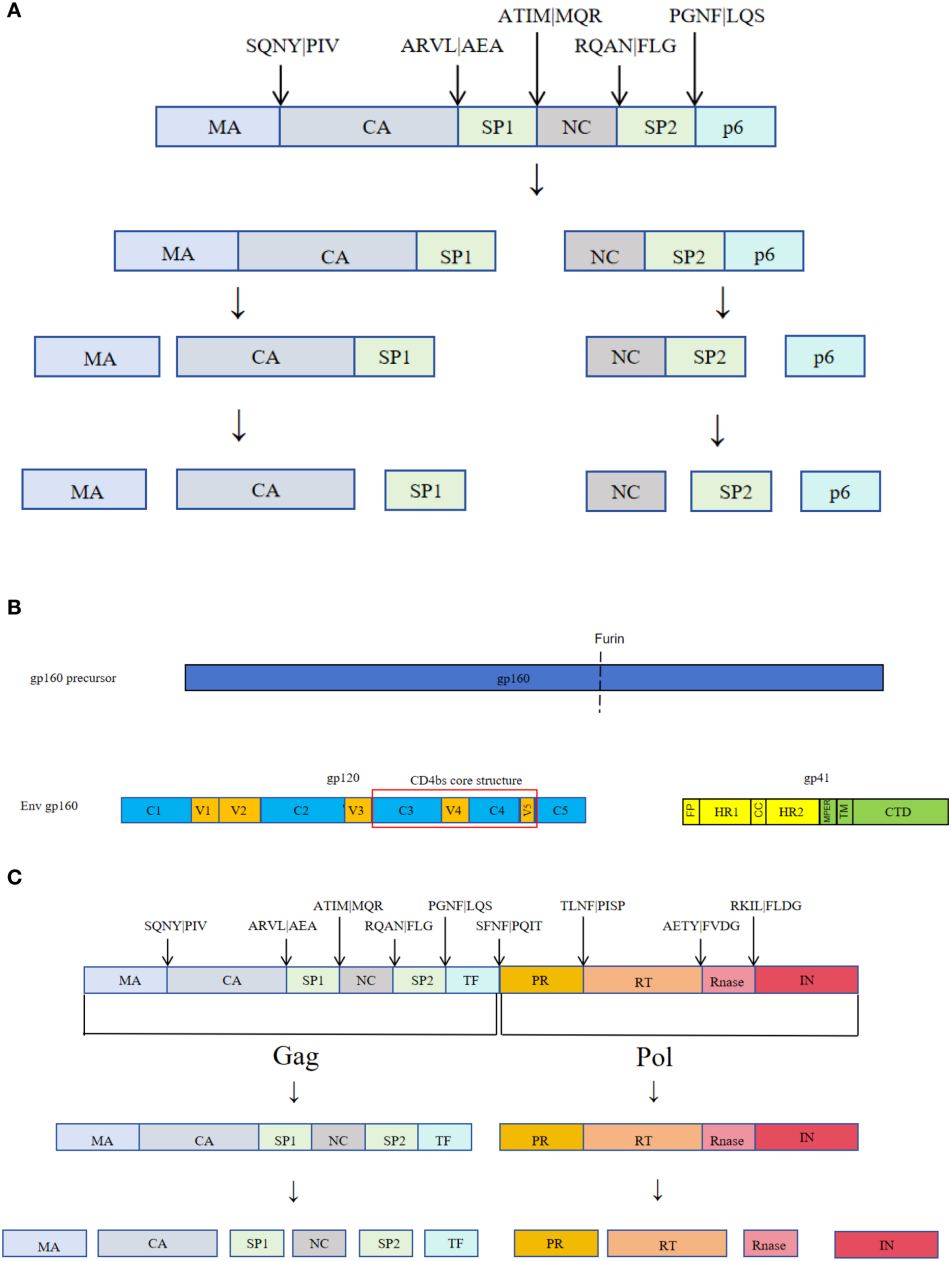
Gag, Gag-Pol and Env polyprotein processing. **(A)** Proteolytic processing starts at the SP1–NC junction at the RQA|NFLG cleavage site, then proceeds to the MA–CA and SP2–p6 junctions at SQNY|PIV and PGNF|LQS, respectively. Subsequently, the spacer peptides SP1 and SP2 are trimmed from CA and NC at ARVL|AEA and RQAN|FLG. **(B)** The HIV-1 Gag–Pol polyprotein is generated by a programed −1 ribosomal frameshift at the NC/SP1 junction, placing the p6 region into an alternative reading frame to form the transframe domain (TF/p6*) upstream of Pol. Maturation is initiated by auto-processing at the TF|PR site (SFNF|PQIT), catalyzed by the weakly active protease precursor. This cleavage separates the N-terminal Gag-derived segment (MA–CA–SP1–NC–SP2–TF) from the Pol enzymes (PR–RT–RNase H–IN), releasing PR to drive subsequent ordered processing. **(C)** The Env protein is initially synthesized as a precursor, gp160 (a glycoprotein with an apparent molecular weight of 160 kDa), which undergoes processing by Furin. Subsequently, gp160 is cleaved by a cellular furin-like protease into two subunits: the receptor-binding subunit gp120 and the fusion subunit gp41.

Gag (Group-specific antigen) is an HIV-1 polyprotein essential for viral structure and infectivity. The Gag precursor (p55) consists of the matrix protein (MA/p17), capsid protein (CA/p24), nucleocapsid protein (NC/p7), and the p6 domain, each of which performs distinct functions (3) (**Figure 1A**).

MA is composed of an N-terminal globular head and a C-terminal tail (4). Functionally, MA directs Gag-Pol and PR55Gag to the plasma membrane (3). This process is governed by two key chemical signals: myristoylation and interaction with the plasma membrane-specific phospholipid phosphatidylinositol-4,5-bisphosphate (PIP2) (5). During viral entry, myristoylation occurs first, in which an N-myristoyltransferase catalyzes the formation of a covalent bond between myristic acid and the MA N-terminal domain (4). This modification increases the affinity of MA for the plasma membrane, helping to target the protein to membrane structures. The second signal involves electrostatic interactions between basic residues (HBR) in MA and negatively charged PIP2 in the plasma membrane (5). Following myristic acid exposure, insertion into the lipid bilayer occurs. During the transition from sequestration to exposure, the PIP2 recognition site (30KLKH34) sequesters PIP2 fatty acids into a hydrophobic pocket. The interactions between the plasma membrane and myristic acid subsequently promote the formation of a bidirectional lipid bilayer (6, 7).

In mature virions, HIV-1 capsid protein oligomerizes to form a protective shell around the viral RNA and core-associated proteins (8). CA consists of two domains: the N-terminal domain (CANTD_1-149_) and the C-terminal domain (CACTD_150-231_), connected by a flexible linker (9). The capsid encloses viral RNA and core proteins, shielding them from antiviral factors and cellular sensors of innate immunity (10, 11). Beyond its structural role, CA participates in multiple stages of the viral replication cycle, including reverse transcription (RT), cytoplasmic transport via microtubules, uncoating of the viral pre-integration complex, nuclear import, integration, and viral assembly (12, 13).

Comprising 55 amino acids, NC contains two highly conserved zinc finger motifs (Cys-X2-Cys-X4-His-X4-Cys) (14). It is responsible for the specific recognition and selective packaging of the viral packaging signal (Psi, Ψ) located in the 5′-leader region of the viral genome, enabling the incorporation of two copies of genomic RNA into nascent virions amid a vast excess of cellular RNA (15). Following viral maturation and release from the cell, NCp7 is liberated through proteolytic cleavage of the Gag polyprotein by the viral protease (16).

p6 domain contains PTAP and YPXL protein interaction motifs, which recruit the host endosomal sorting complex required for transport (ESCRT) to facilitate the budding of immature virions from the host cell (17). The YPXL (Tyr-Pro-X-Leu) motif binds to the cellular factor ALG-2-interacting protein X (ALIX), while PTAP interacts with the host protein tumor susceptibility gene 101 (TSG101) (18). The four PTAP residues contact the N-terminal ubiquitin E2 variant (UEV) domain of TSG101 (19). Thus, p6 can be regarded as a viral mimic that recruits cellular ESCRT-I machinery in HIV-1 (20). Although earlier studies indicated that the NC domain of the precursor PR55 Gag protein primarily governs genomic RNA selection by recognizing the gag gene initiation sequence and the Ψ packaging signal, recent evidence suggests that the p6 domain also modulates the RNA-binding specificity of PR55 (21).

### 2.2 Pol protein

The HIV-1 Pol protein is a core functional protein essential for viral replication. The Gag-to-Gag-Pol output from the same full-length viral mRNA is set by a programed ribosomal frameshift (22). Approximately 5–10% of ribosomes initiating translation on the full-length vRNA shift into the −1 reading frame via a programed−1 ribosomal frameshift, resulting in Gag-Pol rather than Gag (23). It is produced by the proteolytic cleavage of the Gag-Pol precursor polyprotein. This multifunctional enzyme consists of three distinct domains: protease (PR), reverse transcriptase (RT) and integrase (IN) (**Figure 1B**). The PR domain is responsible for cleaving viral polyprotein precursors, the RT domain facilitates the reverse transcription of viral RNA into DNA, and the IN domain catalyzes the integration of the viral DNA into the host genome. Through their coordinated actions, these three domains work together to complete the HIV-1 replication cycle.

PR consists of two identical subunits and belongs to the aspartyl protease family. Each subunit contains a conserved catalytic triad, Asp-Thr/Ser-Gly. Following viral maturation, PR precisely recognizes and hydrolyzes specific peptide bonds within precursor proteins. It sequentially cleaves the Gag and Gag-Pol precursors, thereby processing them into mature, functional viral proteins (22). This process is critical for the formation of infectious viral particles, making PR an important target for antiretroviral drug development.

RT is a heterodimer consisting of p66 and p51 subunits, derived from the proteolytic processing of the Pol polyprotein precursor. Initial homodimeric p66 molecules undergo conformational rearrangement, where one subunit retains full enzymatic activity while the other (p66’) provides structural support. Subsequent cleavage of the C-terminal RNase H domain in the p66’ subunit yields the 440-amino-acid p51 subunit (22). RT exhibits the canonical “hand” architecture, composed of fingers (residues 1-85, 118-155), palm (86-117, 156-236), thumb (237-318), and connection (319-426) subdomains. Although the p51 subunit shares the same primary domain organization as p66, their distinct spatial arrangements result in an overall asymmetric heterodimeric structure (24).The enzyme engages template/primer substrates via its catalytic triad (D110, D185, D186). The polymerase active site and the RNase H active site within the p66 subunit are spatially separated by approximately 18 base pairs. As a multifunctional enzyme, the p66 subunit possesses both DNA-dependent DNA polymerase activity and RNase H activity. The conserved YMDD motif constitutes a critical part of the catalytic center, responsible for DNA synthesis and subsequent degradation of the RNA template. Notably, the inherent lack of a proofreading function in RT contributes to its high error rate, which is a major factor driving the generation of HIV-1 drug resistance (25).

IN is a polynucleotidyl transferase that catalyzes two sequential enzymatic reactions essential for integration: 3’-processing and strand transfer (26). The enzyme is organized into three conserved structural domains: the N-terminal domain (NTD), which binds Zn²^+^ via an HHCC zinc finger motif (involving His and Cys residues) to form a three-helix bundle; the catalytic core domain (CCD), which adopts an RNase H-like fold and contains the conserved DDE catalytic triad; and the C-terminal domain (CTD), which exhibits an SH3-like fold (27).

During the 3’-processing step, IN hydrolytically removes two nucleotides (typically a conserved CA dinucleotide) from each 3’ end of the viral DNA, preparing the termini for integration. It is noteworthy that processing mechanisms can vary among different retroviruses, with some viruses capable of integration without symmetrical processing at both ends (28). In the subsequent strand transfer reaction, IN utilizes the newly exposed 3’-OH groups to cleave the host DNA via an SN2-type mechanism and covalently link the viral DNA ends, resulting in the characteristic 5-base pair duplication of host DNA flanking the integrated provirus (29). The entire catalytic process is dependent on two Mg²^+^ ions coordinated by the DDE motif. These metal ions participate in activating the nucleophile and stabilizing the reaction transition state, a catalytic mechanism that is highly conserved within the polynucleotidyl transferase superfamily (27, 30).

### 2.3 Env protein

HIV-1 targets CD4+ T cells, macrophages, and dendritic cells, initiating infection via host cell attachment and membrane fusion—a process primarily driven by its envelope glycoprotein (Env).

The Env glycoprotein is initially synthesized as a precursor, gp160, which is subsequently cleaved by a cellular protease into two functional subunits: the surface glycoprotein gp120 and the transmembrane glycoprotein gp41 (**Figure 1C**). Heterodimers of gp120 and gp41 assemble into a trimeric complex, forming the functional unit for infectivity on the surface of the virion (31, 32). While multiple cellular factors can participate in the initial attachment phase, the specific binding of gp120 to the CD4 receptor on the host cell surface is the crucial step that initiates the infection cascade (31).

The binding of gp120 to CD4 induces significant conformational changes within the Env trimer. This structural rearrangement exposes the coreceptor binding site on gp120, allowing it to engage with either the CCR5 or CXCR4 chemokine coreceptor (31). The sequential interaction of gp120 with the primary receptor CD4 and a coreceptor (CCR5 or CXCR4) triggers further major conformational changes in the gp41 subunit. These changes result in the insertion of the gp41 N-terminal fusion peptide into the target cell membrane (33). Finally, gp41 refolds into a highly stable six-helix bundle hairpin structure. This structural transition provides the driving force required for bringing the viral and cellular membranes into close proximity, thereby facilitating their fusion and completing the critical entry step of the viral life cycle (34).

## 3 Research advances in molecular mechanisms and signaling pathways of HIV-1 protein inhibitors

The replication of HIV-1 relies on interactions between various viral proteins and host factors. Inhibitors targeting these proteins have become a core strategy in antiviral therapy. Currently, inhibitors of reverse transcriptase (RT), protease (PR), integrase (IN), and envelope protein (Env) are widely used in clinical practice. The following sections summarize the action targets, molecular mechanisms, and host signaling pathways associated with these inhibitors.

### 3.1 Gag inhibitors

Bevirimat (BVM), the first maturation inhibitor to enter clinical trials, functions by specifically binding to the CA-SP1 junction region. This interaction stabilizes the immature Gag lattice, thereby inhibiting the maturation of viral particles (35). However, its development was discontinued after the emergence of pre-existing CA-SP1 resistance mutations in a subset of patients during clinical trials (36). GSK-3532795 was designed with structural modifications to improve resilience against such resistance, but its Phase II development was terminated due to insufficient efficacy. Subsequently, GS-CA1 and GS-6207 (Lenacapavir) have demonstrated potent antiviral activity. Among these, Lenacapavir has received FDA approval for clinical use (37) (**Table 1**). Notably, lenacapavir is particularly important not only because it targets a novel binding site at the capsid multimer interface, but also because its favorable pharmacokinetic profile enables subcutaneous dosing every 6 months, thereby linking capsid-directed inhibition to modern long-acting treatment strategies (38, 39).

**Table 1:** Antivirals targeting HIV-1 particle maturation and capsid function.

Class	Products	Structure (target pocket/site)	Stage of development	References and/or ClinicalTrials.gov registry numbers
PIs	Saquinavir (SQV)	Small-molecule PI	Approved (treatment)	(128)
	Darunavir (DRV)	HIV-1 protease active site	Approved (standard ART option; usage depends on regimen context)	(71); NCT00258557
MIs	GSK3640254	Gag CA–SP1 (maturation cleavage region)	Phase 2b (reported)	(129); NCT04900038; NCT04493216
	GSK2838232	Gag CA–SP1	Phase 2a (POC reported)	(130); NCT03045861
	GSK3532795	Gag CA–SP1	Phase 2b (reported; development limited by tolerability in studies)	NCT02415595
	Bevirimat (BVM)	Gag maturation (CA–SP1 region)	Phase 2 (historical; not advanced)	NCT01097070
CAIs	Lenacapavir for treatment (HTE/MDR HIV-1)	Capsid (CA) multimer interface pocket	Approved (treatment)	(75); NCT04150068
	Lenacapavir for PrEP	Capsid (CA) pocket	Phase 3 completed; regulatory adoption documented in public health guidance	NCT04994509, NCT04925752; (85, 131)
	Once-yearly Lenacapavi	Capsid (CA) pocket	Phase 1 PK/safety published; Phase 3 registered (PURPOSE 365)	(131); NCT07047716
	Once-weekly oral ISL/LEN (islatravir + lenacapavir)	NRTTI + capsid inhibitor combination	Phase 2 (48-week results reported)	(132); NCT05052996
	Once-weekly oral ISL/LEN (fixed-dose switch; Phase 3)	NRTTI + capsid inhibitor combination	Phase 3 (registered)	NCT06630299

### 3.2 Pol inhibitors

Reverse transcriptase is the key enzyme responsible for converting the HIV-1 RNA genome into cDNA. Its inhibitors are broadly categorized into nucleoside reverse transcriptase inhibitors (NRTIs) and non-nucleoside reverse transcriptase inhibitors (NNRTIs).

#### 3.2.1 Nucleoside reverse transcriptase inhibitors

NRTIs were the first class of drugs approved to treat HIV-1. Resistance to them mainly arises from the high error rate of HIV reverse transcriptase (RT) during replication. The key resistance mechanisms involve either excision, where a mutated RT removes an incorporated NRTI, or discrimination, where mutations alter the dNTP-binding pocket to selectively block NRTI-triphosphate incorporation (4, 40). These mutations typically occur in or near the dNTP binding site, disrupting drug binding or enhancing excision (40).

Clinically used NRTIs fall into two categories. The first includes nucleoside analogs such as zidovudine (AZT) and lamivudine (3TC), which require intracellular phosphorylation to become active. The second includes nucleotide analogs like tenofovir (TFV), which are usually given as prodrugs to improve cellular uptake (25). AZT became the first approved HIV-1 drug in 1987. To date, eight NRTIs have been approved for clinical use, with their efficacy heavily dependent on cellular uptake and phosphorylation efficiency (41). Clinically, several NRTI resistance pathways remain particularly important. M184V/I confers high-level resistance to lamivudine and emtricitabine, K65R is commonly selected under tenofovir pressure and confers reduced susceptibility to tenofovir, and accumulated thymidine analogue mutations can further compromise NRTI susceptibility when combined with other reverse transcriptase mutations (42–44).

#### 3.2.2 Non-nucleoside reverse transcriptase inhibitors

NNRTIs inhibit HIV-1 reverse transcriptase (RT) by binding to a specific, non-catalytic hydrophobic pocket known as the non-nucleoside inhibitor binding pocket (NNIBP). This binding site is located approximately 10 Å from the polymerase active site. The interaction induces conformational changes in RT that disrupt its enzymatic function, thereby inhibiting viral DNA synthesis (40, 45).

Structural studies have elucidated the mechanism of NNRTI action: the induced conformational shift impairs dNTP binding and inhibits polymerization across different nucleic acid substrates, including dsRNA, RNA-DNA hybrids, and dsDNA, next-generation inhibitors of the DAPY class, such as rilpivirine and doravirine, were designed to retain potency against common NNRTI resistance mutations (46).

To date, six NNRTIs have received clinical approval, ranging from first-generation nevirapine to the recently approved doravirine. NNRTIs exhibit high selectivity for HIV-1 RT, showing no activity against HIV-2 RT and generally low cellular toxicity. This intrinsic resistance is mainly due to sequence and structural differences in the HIV-2 NNRTI-binding pocket that impair NNRTI binding (2). However, the structural flexibility of the NNIBP also contributes to a high propensity for the development of drug resistance. Clinically, resistance to first-generation NNRTIs is still commonly associated with key substitutions such as K103N and Y181C, whereas rilpivirine resistance is frequently linked to mutations including E138K, K101E, and L100I. Newer NNRTIs such as doravirine retain improved activity against some common NNRTI-resistant variants, but cross-resistance may still emerge in selected settings, particularly after extensive prior NNRTI exposure (47, 48).

### 3.3 Integrase inhibitors

The integration of the HIV-1 genome into host chromatin is regulated by cellular cofactors. The viral integrase (IN) interacts with the host protein LEDGF/p75, which facilitates the targeted insertion of viral DNA into transcriptionally active regions of the genome (49). LEDGF/p75 contains an IN-binding domain at its C-terminus, while its N-terminal PWWP domain recognizes histone H3 lysine 36 trimethylation (H3K36me3) to mediate chromatin association (50, 51). Studies indicate that when LEDGF/p75 is knocked down, its homolog HRP-2 can partially compensate for its function (52). Notably, even upon dual knockout of LEDGF/p75 and HRP-2, HIV-1 integration does not become entirely random, suggesting the involvement of additional cellular factors. Furthermore, disruption of the IN-LEDGF/p75 interaction—either through LEDGF/p75 depletion or via small-molecule inhibitors known as LEDGINs—results in a shift of integration sites toward more inner nuclear compartments (49).

These findings further support integrase-centered processes as therapeutically relevant targets. Clinically, the most established integrase-directed agents are integrase strand transfer inhibitors (INSTIs), which block the strand transfer step of viral DNA integration. Resistance to first-generation INSTIs, such as raltegravir and elvitegravir, most commonly follows the Y143, Q148, and N155 pathways, whereas second-generation agents, including dolutegravir, bictegravir, and cabotegravir, generally retain a higher genetic barrier, although mutations such as R263K, G118R, or Q148-pathway variants with accessory substitutions may still emerge under treatment pressure (53, 54).

### 3.4 Env inhibitors

Env exhibits greater variability than viral enzymes like reverse transcriptase. However, its conformational changes during fusion expose highly conserved functional regions, making it a suitable target for broad-spectrum fusion inhibitors (**Table 2**). Unlike enzyme inhibitors that must enter the cell, Env inhibitors act at the cell surface. With no human homolog, they offer greater specificity and a favorable safety profile.

**Table 2:** Env-directed entry inhibitors (HIV-1).

Class	Products	Target site	Study endpoints being evaluated	References and/or ClinicalTrials.gov registry numbers
Single	VRC01	CD4bs	Efficacy to prevent sexual transmission	NCT02568215; NCT02716675;
	PGT121.414.LS	V3 loop	Safety and PK in HIV- and HIV+ adults and exposed infants	(133)
	PGDM1400LS	V2 apex	Safety and PK in HIV- and HIV+ adults and exposed infants	(134)
	CAP256V2LS	V2 apex	Safety and PK in HIV- and HIV+ adults and exposed infants	NCT04408963
	10E8VLS	MPER	Safety and PK in HIV- and HIV+ adults and exposed infants	NCT03565315; (135)
	VRC07-523LS	CD4bs	Safety and PK in HIV- and HIV+ adults and exposed infants	(136)
	N6LS (VH3810109)	CD4bs	Safety and PK in HIV- and HIV+ adults and exposed infants	(137)
Combo	Teropavimab (3BNC117-LS)+ Zinlirvimab (10-1074-LS) + Lenacapavir	CD4bs/V3 loop	Safety and PK in HIV- and HIV+ adults and exposed infants/Maintenance of suppression as long-acting regimen	(77)
	Teropavimab (3BNC117-LS) + Zinlirvimab (10-1074-LS) regimen	CD4bs/V3 loop	Safety and PK in HIV- and HIV+ adults and exposed infants/Maintenance of suppression as long-acting regimen	NCT05729568
	10-1074-LS ± 3BNC117-LS	V3 loop	Safety and PK in HIV- and HIV+ adults and exposed infants	NCT03554408
	3BNC117-LS + 10-1074-LS (RIO)	CD4bs/V3 loop	Antiviral activity during viremia and analytical treatment interruption	NCT04319367
	3BNC117-LS + 10-1074-LS (A5364)	CD4bs/V3 loop	Antiviral activity during viremia and analytical treatment interruption	NCT05079451
	VRC07-523LS + CAP256V2LS	CD4bs/V2 apex	Antiviral activity during viremia and analytical treatment interruption	NCT05281510
	VRC07-523LS + PGT121.414.LS + PGDM1400LS	CD4bs/V3 loop/V2 apex	Efficacy to prevent sexual transmission/Safety and PK in HIV- and HIV+ adults and exposed infants	NCT06812494
	TMB-365 + TMB-380	CD4/CD4bs	Maintenance of suppression as long-acting regimen/Safety and PK in HIV- and HIV+ adults and exposed infants	NCT07215468
Multi-specific	10E8.4/iMab bispecific	MPER/CD4 (± CD4bs)	Antiviral activity during viremia and analytical treatment interruption/Safety and PK in HIV- and HIV+ adults and exposed infants	NCT05890963
	SAR441236	CD4bs/V2 apex/MPER	Safety and PK in HIV- and HIV+ adults and exposed infants/Antiviral activity during viremia and analytical treatment interruption	NCT03705169
	CAP256J3LS	V2 apex	Safety and PK in HIV- and HIV+ adults and exposed infants	NCT06585891
Gene transfer	rAAV1-PG9DP	V2 apex	Safety and PK in HIV- and HIV+ adults and exposed infants	(138)

#### 3.4.1 Protein-/peptide-based fusion inhibitors

Ibalizumab is a monoclonal antibody targeting the CD4 receptor. It binds to the CD4 D1-D2 junction region without directly blocking the gp120 binding site. This binding induces conformational changes and steric hindrance, preventing HIV-1 from interacting with CD4. Its heavy-chain CDR-H3 region mimics the key CD4 residue Phe43, occupying the conserved hydrophobic pocket on gp120 to effectively inhibit viral attachment. Approved by the FDA in March 2018 for multidrug-resistant (MDR) HIV-1, its Phase III trials demonstrated significant efficacy (55). Although high cost and resistance risk—linked to V5-loop glycan loss—remain challenges, it is a key option for MDR patients (56).Although ibalizumab is currently approved for multidrug-resistant HIV-1, its CD4-directed post-attachment mechanism, rather than direct binding to a variable viral Env epitope, may explain why HIV-2 isolates can remain susceptible *in vitro*; however, clinical evidence in HIV-2 is still limited (57).

Early soluble CD4 showed limited efficacy against primary HIV-1 isolates, dampening interest in decoy-receptor strategies. However, studies on CD4–gp120 interaction revealed that CD4 binds gp120 via its Phe43 residue, which inserts into a conserved cavity between the inner and outer domains of gp120 (45). This led to the design of CD4-mimetic miniproteins, such as M48U12, which accurately mimic CD4’s gp120-binding properties. Their ability to engage the Phe43 cavity enables potent viral inhibition at low concentrations.

The formation of the gp41 six-helix bundle structure represents a critical step in HIV-1 infection (58, 59) This mechanism has enabled the development of several peptide-based inhibitors. Enfuvirtide (T-20), derived from the gp41 HR2 region, was the first fusion inhibitor approved by the FDA. It functions by competitively binding to the HR1 domain, thereby preventing the formation of the six-helix bundle (60). Importantly, enfuvirtide should not be directly extrapolated to HIV-2, because HIV-2 shows markedly reduced susceptibility to this fusion inhibitor. This difference is thought to result from natural sequence variation in the gp41 heptad-repeat target region, which can weaken inhibitor-target interactions and reduce the ability of T-20 to block six-helix bundle formation (2). However, its clinical application is constrained by the need for refrigeration, twice-daily subcutaneous administration, and a tendency to induce drug resistance (61). In contrast, Albuvirtide (FB006M), an HR2 peptide derivative approved in China, has been modified with maleimidopropionic acid to enable binding to human serum albumin. This modification not only significantly enhances its resistance to proteolytic degradation but also extends its half-life to approximately ten times that of enfuvirtide (62), indicating a more favorable clinical application profile.

#### 3.4.2 Small-molecule fusion inhibitors

CCR5 serves as a key therapeutic target, with the CCR5Δ32 mutation conferring natural resistance to HIV-1 infection. Maraviroc, approved by the FDA in 2007, specifically binds to the CCR5 transmembrane domain and competitively disrupts gp120 engagement, thereby inhibiting viral entry. Because maraviroc is active only against CCR5-tropic HIV-1, tropism testing is mandatory before prescription to confirm the absence of CXCR4-using or dual/mixed-tropic virus (63). For HIV-2, maraviroc may be relevant only for R5-tropic viruses. *In vitro* studies suggest that HIV-2 R5 isolates can remain susceptible to maraviroc, but the required inhibitory concentrations may be higher than those for HIV-1, and current clinical evidence remains limited (2).

High-throughput screening identified NBD-11021, an optimized analog of the CD4-mimetic NBD-556, with improved antiviral activity and no enhancement of infection in CD4-negative/CCR5-positive cells. Another class is represented by fostemsavir (BMS-663068), a prodrug of the active attachment inhibitor temsavir, which binds directly to gp120 near the CD4-binding site and prevents the conformational changes required for viral attachment. Fostemsavir was approved in 2020 for heavily treatment-experienced adults with multidrug-resistant HIV-1 (38, 64). Another class, represented by BMS analogs such as BMS-663068 (approved in 2018 for multidrug-resistant HIV-1), binds to a pocket near the CD4-binding site and blocks gp120 conformational changes required for viral attachment (38, 65, 66).

The hydrophobic pocket formed by HR1 helices in gp41 has been exploited for inhibitor design. ADS-J1, identified via computational docking, shows inhibitory activity at low micromolar levels (67). Similarly, 5M038 inhibits viral fusion in the micromolar range. Other candidates such as NB-2, NB-64, and derivatives including A12 and NB-293 require further mechanistic validation.

The membrane-proximal external region (MPER) of gp41 is a conserved epitope recognized by broadly neutralizing antibodies such as 2F5, 4E10, Z13e1, 10E8 and DH511 (68). The small molecule S2C3, a dequalinium derivative served as MPER-targeting compound, broadly inhibits HIV-1, HIV-2 and SIV entry (69). Structural studies using NMR on MPER-TMD in bicelles confirmed that S2C3 binds to a hydrophobic pocket formed by residues from adjacent MPER protomers (69). S2C3 stabilizes the prefusion conformation of Env, preventing CD4-induced conformational changes and blocking soluble CD4 binding to cell-surface Env. These findings highlight the potential of MPER-targeting compounds as novel fusion inhibitors (69).

## 4 Current therapeutic strategies and challenges in HIV-1 management

**Figure 2:**
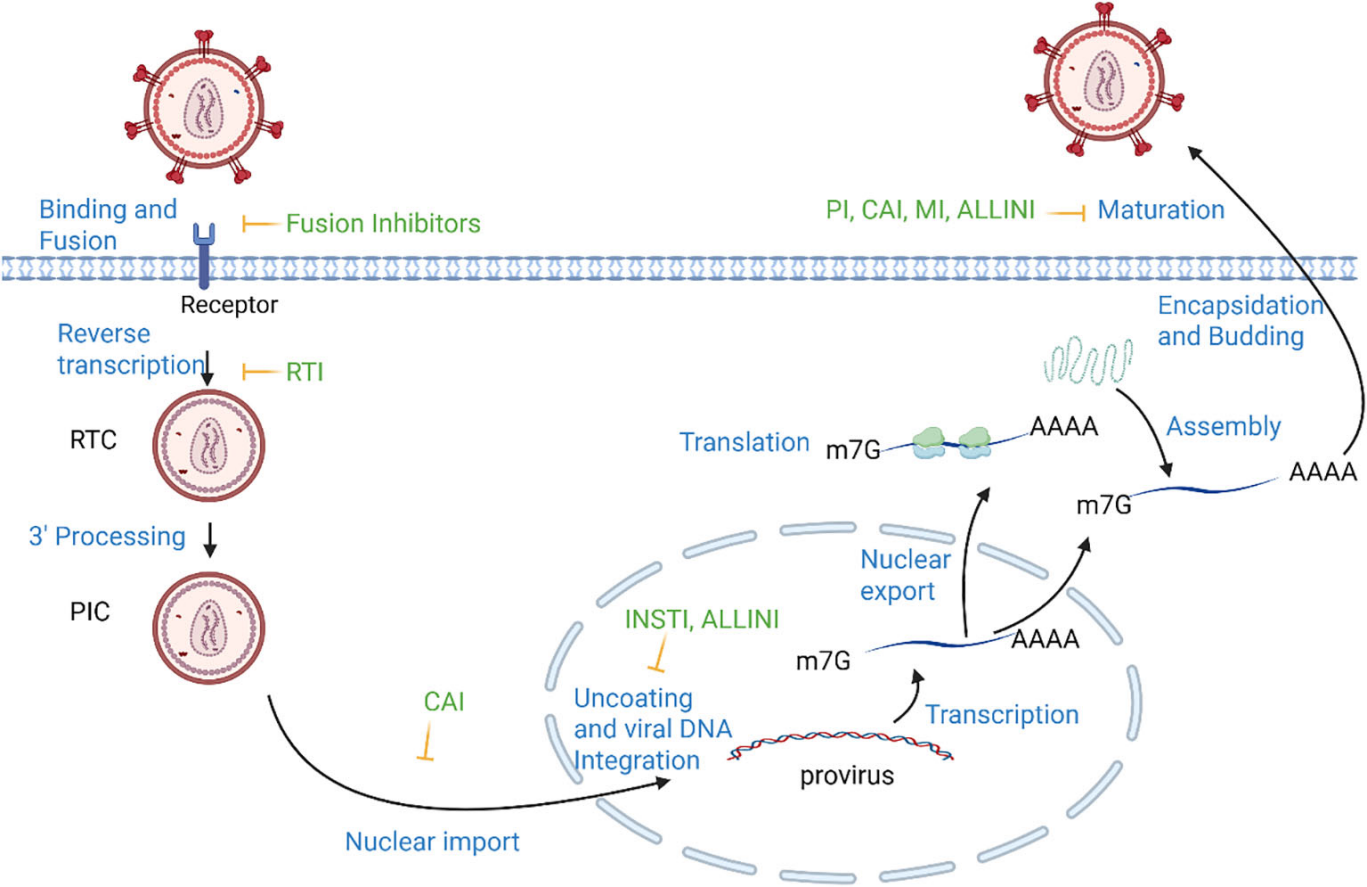
HIV-1 replication cycle and the indication of drugs target structural proteins. Upon entering the target cell, reverse transcriptase (RT) transcribes genomic RNA into a reverse transcription complex (RTC). Through IN processing of viral DNA ends, a proviral integration complex (PIC) is generated, which can integrate endogenous DNA prepared by reverse transcription into the DNA. After nuclear entry, the provirus serves as a template for transcription, producing mRNAs that are used to translate viral proteins and assemble with viral proteins into the viral genome. Subsequently, budding occurs, releasing mature HIV-1 virus particles.

Significant advances in HIV-1 treatment have transformed AIDS from a fatal disease into a manageable chronic condition (70). The current standard of care is highly active antiretroviral therapy, which typically combines multiple drug classes, including nucleoside reverse transcriptase inhibitors (Tenofovir, Lamivudine, Abacavir), non-nucleoside reverse transcriptase inhibitors (Rilpivirine), and integrase strand transfer inhibitors (Dolutegravir) (71, 72). This combination effectively suppresses viral replication, restores immune function, and prolongs life expectancy (**Figure 2**).

Recent innovations have further optimized treatment paradigms, particularly through the development of long-acting treatment strategies. Cabotegravir plus rilpivirine provides a maintenance option for virologically suppressed individuals and has shown durable efficacy through 96 and 152 weeks in ATLAS-2M (73, 74). Lenacapavir, a long-acting capsid inhibitor administered every 6 months, has also shown sustained antiviral activity in heavily treatment-experienced individuals with multidrug-resistant HIV-1 when combined with an optimized background regimen (75, 76). Investigational regimens combining lenacapavir with broadly neutralizing antibodies have also shown promise, although they remain under clinical evaluation (77).

Despite these advances, drug resistance remains a major clinical challenge and is closely linked to long-term treatment success. Baseline genotypic resistance testing remains important for identifying transmitted resistance, while resistance testing at virologic failure is essential for distinguishing true resistance from suboptimal adherence and for guiding regimen modification (78, 79). Persistent low-level viraemia should not be overlooked because it may indicate emerging resistance or adherence problems (80). By contrast, non-suppressible viraemia may arise from clonally expanded infected cells without evidence of ongoing viral evolution (81). When plasma HIV-1 RNA levels are too low for successful RNA genotyping, proviral HIV-1 DNA genotyping may provide complementary information, although its interpretation requires caution because archived, defective, or hypermutated sequences can be detected (82).

The therapeutic arsenal continues to expand with novel agents, including post-attachment inhibitor (Ibalizumab) targeting the viral envelope, new capsid inhibitors (Lenacapavir), and improved fixed-dose combinations (Biktarvy), all contributing to enhanced treatment adherence and patient quality of life (75, 83, 84).

A major obstacle to curing HIV-1 is the establishment of a latent viral reservoir. Following initial infection, the HIV-1 genome integrates into the host cell DNA. While the immune system rapidly clears most infected cells, a small fraction enters a state of latency. In these cells, the integrated provirus remains transcriptionally silent, does not produce viral proteins, and thereby evades immune detection, allowing the infected cells to persist long-term (85–88). These latently infected cells, residing in tissues such as the central nervous system and gut-associated lymphoid tissue, constitute a stable reservoir that is not eliminated by current antiretroviral therapy and is considered the primary barrier to an HIV-1 cure (89–91).

Current cure strategies primarily focus on targeting this reservoir. Two major therapeutic approaches are under investigation. One approach is latency reversal followed by clearance of reactivated cells, in which latency-reversing agents (LRAs), such as histone deacetylase inhibitors and PKC agonists, are used to reactivate viral gene expression in latently infected cells. Representative LRA classes include histone deacetylase inhibitors (HDACis), such as vorinostat and panobinostat, and protein kinase C (PKC) agonists, such as bryostatin and ingenol-derived compounds, which promote HIV transcription through distinct epigenetic or signaling mechanisms (92, 93). This approach is intended to expose infected cells to the immune system and may be combined with immune-based interventions, including broadly neutralizing antibodies, to enhance clearance of reactivated cells, although the ability of current LRAs to reduce the reservoir *in vivo* remains limited (94, 95). Conversely, another approach is durable proviral silencing, which seeks to maintain deep latency or long-term viral silencing using epigenetic modifiers such as dCA, thereby maintaining the provirus in a transcriptionally inactive state and preventing future reactivation (96, 97). A representative agent in this context is the Tat inhibitor didehydro-cortistatin A (dCA), which suppresses Tat-dependent transcription and promotes a more stable silencing state, thereby reducing the likelihood of future viral reactivation (98, 99).

Cell and gene therapies have emerged as a pivotal direction in HIV-1 cure research, focusing primarily on CRISPR-based gene editing and CCR5 gene modification. Editing the CCR5 gene using tools like CRISPR-Cas9 aims to disrupt this coreceptor and block viral entry into host cells (96, 100, 101). This approach is supported by the cases of the “Berlin patient” and “London patient,” who achieved sustained HIV-1 remission following hematopoietic stem cell transplantation from donors with a natural CCR5-Δ32 mutation. While such a procedure can confer complete resistance to CCR5-tropic HIV-1 strains, its applicability is limited. It is ineffective against CXCR4-tropic viruses and carries significant risks associated with allogeneic hematopoietic stem cell transplantation (HSCT) and prolonged immunosuppression, precluding its widespread use (102–105).

Current gene therapy strategies are evolving towards greater diversity. One avenue involves the development of CAR-T cells engineered to specifically recognize and clear HIV-1-infected cells. Another utilizes novel delivery systems to enable the sustained *in vivo* expression of antiviral agents, such as broadly neutralizing antibodies and CD4 mimetics (106–109). The most advanced strategy involves using gene-editing tools like CRISPR-Cas9 to directly target and excise the integrated provirus. However, this approach faces significant challenges related to target specificity, sequence heterogeneity of the virus, and potential off-target effects (110–114).

The application of CAR-T cell therapy for HIV-1 presents unique considerations. During suppressive antiretroviral therapy (ART), viral antigen levels are often too low to drive adequate CAR-T cell activation and expansion. Researchers are investigating strategies to overcome this, including analytical treatment interruption to increase antigen exposure or the use of more potent latency-reversing agents to enhance viral protein expression (115–118). Recent studies in non-human primates suggest that novel adjuvants can effectively expand CAR-T cells even under conditions of low antigen burden, offering a promising direction for optimizing this therapeutic modality. Collectively, these advances are propelling HIV-1 gene therapy from proof-of-concept towards potential clinical translation (119).

While combination antiretroviral therapy (cART) effectively suppresses plasma viral loads below detection limits, its principal limitation is rapid viral rebound upon treatment cessation (120–122). To address this, strategies include strengthening drug resistance monitoring to guide individualized therapy. The use of newer agents with high genetic barriers to resistance, such as the integrase inhibitor dolutegravir and the capsid inhibitor lenacapavir, can reduce the risk of resistance development. Furthermore, optimized combination regimens (Tenofovir + Lamivudine + Dolutegravir) help overcome cross-resistance and minimize the accumulation of resistance mutations (123, 124). In parallel, vaccine-related approaches remain an important area of future development and are discussed further in the following section, particularly in the context of next-generation immunogen design and mRNA-based platforms (76, 125–127).

## 5 Future research directions and perspectives

While combination antiretroviral therapy (cART) has profoundly improved the life expectancy and quality of life for people living with HIV, significant challenges remain. These include the persistence of viral reservoirs, the rising threat of drug resistance, and the long-term toxicity associated with lifelong therapy. This review outlines several promising directions for future research and therapeutic development.

This involves targeting key viral lifecycle proteins, such as CA, IN, and RT, to develop new inhibitors that overcome existing drug resistance. This includes optimizing broadly neutralizing antibodies (bNAbs)—using structural biology to improve their potency and breadth against diverse HIV-1 strains and exploring their combination with immunomodulators. Furthermore, developing long-acting sustained-release formulations can reduce dosing frequency and improve patient adherence.

For CAR-T/TCR-T cell therapies, it is necessary to refine T-cell engineering techniques to enable durable recognition and clearance of latently infected cells while protecting the engineered cells from HIV-1 infection. For CRISPR/Cas9 gene-editing technology, enhancing its precision in targeting integrated proviral DNA or host dependency factors (e.g., CCR5) is crucial to achieve the goal of reservoir elimination.

Combining cART with immunotherapy and gene therapy may enhance overall treatment efficacy. Concurrently, advancing personalized precision medicine—tailoring combination regimens based on individual viral reservoir characteristics and immune status—is key to improving the likelihood of a cure.

Building on the success of COVID-19 vaccines, developing preventive or therapeutic HIV-1 mRNA vaccines to induce stronger humoral and cellular immune responses is a promising avenue. Research into nanodelivery systems to improve the delivery efficiency of drugs or gene-editing tools to viral reservoir sites (e.g., the brain, gut-associated lymphoid tissue) is also critical.

## 6 Discussion

This narrative review summarizes the critical roles of HIV-1 structural proteins and corresponding therapeutic targeting strategies. The structural proteins Gag, Pol, and Env play indispensable roles in the viral replication cycle, and their precise molecular mechanisms provide vital targets for antiviral drug design. Although existing antiretroviral drugs have achieved notable success in suppressing viral replication, persistent issues remain concerning drug resistance and therapeutic precision.

Future advancements necessitate a multidisciplinary approach, integrating progress from molecular biology, immunology, genetic engineering, and clinical medicine. While achieving a complete sterilizing cure for HIV-1 remains a formidable challenge, the continuous breakthroughs in novel therapies—such as long-acting antibodies, gene editing, and immune modulation—make the prospect of a functional cure (long-term viral control without lifelong medication) an increasingly attainable goal in the field of HIV-1 treatment.

## References

[1] Sumner C Ono A . The “basics” of HIV-1 assembly. PloS Pathog. (2024) 20:e1011937. doi: 10.1371/journal.ppat.1011937. PMID: 38300900 PMC10833515

[2] Moranguinho I Taveira N Bártolo I . Antiretroviral treatment of HIV-2 infection: Available drugs, resistance pathways, and promising new compounds. Int J Mol Sci. (2023) 24(6):5905. doi: 10.3390/ijms24065905. PMID: 36982978 PMC10053740

[3] Samsudin F Gan S Bond P . The impact of Gag non-cleavage site mutations on HIV-1 viral fitness from integrative modelling and simulations. Comput Struct Biotechnol J. (2021) 19:330–42. doi: 10.1016/j.csbj.2020.12.022. PMID: 33425260 PMC7779841

[4] Bou-Nader C Muecksch F Brown J Gordon J York A Peng C . HIV-1 matrix-tRNA complex structure reveals basis for host control of Gag localization. Cell Host Microbe. (2021) 29:1421–1436.e7. doi: 10.1016/j.chom.2021.07.006. PMID: 34384537 PMC8650744

[5] Murphy R Saad J . The interplay between HIV-1 Gag binding to the plasma membrane and Env incorporation. Viruses. (2020) 12(5):548. doi: 10.3390/v12050548. PMID: 32429351 PMC7291237

[6] Samal A Green T Saad J . Atomic view of the HIV-1 matrix lattice; implications on virus assembly and envelope incorporation. Proc Natl Acad Sci USA. (2022) 119:e2200794119. doi: 10.1073/pnas.2200794119. PMID: 35658080 PMC9191676

[7] Stacey J Hrebík D Nand E Shetty S Qu K Boicu M . The conserved HIV-1 spacer peptide 2 triggers matrix lattice maturation. Nature. (2025) 640:258–64. doi: 10.1038/s41586-025-08624-9. PMID: 40011770 PMC11964938

[8] Troyano-Hernáez P Reinosa R Holguín Á . HIV capsid protein genetic diversity across HIV-1 variants and impact on new capsid-inhibitor lenacapavir. Front Microbiol. (2022) 13:854974. doi: 10.3389/fmicb.2022.854974. PMID: 35495642 PMC9039614

[9] McFadden W Casey-Moore M Bare G Kirby K Wen X Li G . Identification of clickable HIV-1 capsid-targeting probes for viral replication inhibition. Cell Chem Biol. (2024) 31:477–486.e7. doi: 10.1016/j.chembiol.2024.02.012. PMID: 38518746 PMC11257216

[10] Burdick R Morse M Rouzina I Williams M Hu W Pathak V . HIV-1 uncoating requires long double-stranded reverse transcription products. Sci Adv. (2024) 10:eadn7033. doi: 10.1126/sciadv.adn7033. PMID: 38657061 PMC11042746

[11] Scott T Arnold L Powers J McCann D Rowe A Christensen D . Cell-free assays reveal that the HIV-1 capsid protects reverse transcripts from cGAS immune sensing. PloS Pathog. (2025) 21:e1012206. doi: 10.1371/journal.ppat.1012206. PMID: 39874383 PMC11793794

[12] Dharan A Bachmann N Talley S Zwikelmaier V Campbell E . Nuclear pore blockade reveals that HIV-1 completes reverse transcription and uncoating in the nucleus. Nat Microbiol. (2020) 5:1088–95. doi: 10.1038/s41564-020-0735-8. PMID: 32483230 PMC9286700

[13] Toccafondi E Lener D Negroni M . HIV-1 capsid core: A bullet to the heart of the target cell. Front Microbiol. (2021) 12:652486. doi: 10.3389/fmicb.2021.652486. PMID: 33868211 PMC8046902

[14] Wang Y Guo C Wang X Xu L Li R Wang J . The zinc content of HIV-1 NCp7 affects its selectivity for packaging signal and affinity for stem-loop 3. Viruses. (2021) 13(10):1922. doi: 10.3390/v13101922. PMID: 34696351 PMC8540335

[15] Ding P Kharytonchyk S Waller A Mbaekwe U Basappa S Kuo N . Identification of the initial nucleocapsid recognition element in the HIV-1 RNA packaging signal. Proc Natl Acad Sci USA. (2020) 117:17737–46. doi: 10.1073/pnas.2008519117. PMID: 32647061 PMC7395439

[16] Tabler C Wegman S Chen J Shroff H Alhusaini N Tilton J . The HIV-1 viral protease is activated during assembly and budding prior to particle release. J Virol. (2022) 96:e0219821. doi: 10.1128/jvi.02198-21. PMID: 35438536 PMC9093094

[17] Bernacchi S . Visualization of retroviral Gag-genomic RNA cellular interactions leading to genome encapsidation and viral assembly: An overview. Viruses. (2022) 14(2):324. doi: 10.3390/v14020324. PMID: 35215917 PMC8876502

[18] Meusser B Purfuerst B Luft F . HIV-1 Gag release from yeast reveals ESCRT interaction with the Gag N-terminal protein region. J Biol Chem. (2020) 295:17950–72. doi: 10.1074/jbc.RA120.014710. PMID: 32994219 PMC7939435

[19] Murciano-Calles J Rodríguez-Martínez A Palencia A Andújar-Sánchez M Iglesias-Bexiga M Corbi-Verge C . Phage display identification of high-affinity ligands for human TSG101-UEV: A structural and thermodynamic study of PTAP recognition. Int J Biol Macromol. (2024) 274:133233. doi: 10.1016/j.ijbiomac.2024.133233. PMID: 38901510

[20] Montero F Parra-López M Rodríguez-Martínez A Murciano-Calles J Buzon P Han Z . Exploring the druggability of the UEV domain of human TSG101 in search for broad-spectrum antivirals. Protein Sci. (2025) 34:e70005. doi: 10.1002/pro.70005. PMID: 39724449 PMC11670305

[21] Lei X Gonçalves-Carneiro D Zang T Bieniasz P . Initiation of HIV-1 Gag lattice assembly is required for recognition of the viral genome packaging signal. Elife. (2023) 12:e83548. doi: 10.7554/eLife.83548. PMID: 36688533 PMC9908077

[22] Harrison J Passos D Bruhn J Bauman J Tuberty L DeStefano J . Cryo-EM structure of the HIV-1 Pol polyprotein provides insights into virion maturation. Sci Adv. (2022) 8:eabn9874. doi: 10.1126/sciadv.abn9874. PMID: 35857464 PMC9258950

[23] Kibe A Buck S Gribling-Burrer A Gilmer O Bohn P Koch T . The translational landscape of HIV-1 infected cells reveals key gene regulatory principles. Nat Struct Mol Biol. (2025) 32:841–52. doi: 10.1038/s41594-024-01468-3. PMID: 39815046 PMC12086091

[24] Kirby T Gabel S DeRose E Perera L Krahn J Pedersen L . Targeting the structural maturation pathway of HIV-1 reverse transcriptase. Biomolecules. (2023) 13(11):1603. doi: 10.3390/biom13111603. PMID: 38002285 PMC10669680

[25] Singh A Das K . Insights into HIV-1 reverse transcriptase (RT) inhibition and drug resistance from thirty years of structural studies. Viruses. (2022) 14(5):1027. doi: 10.3390/v14051027. PMID: 35632767 PMC9148108

[26] Li M Yang R Chen X Wang H Ghirlando R Dimitriadis E . HIV-1 integrase assembles multiple species of stable synaptic complex intasomes that are active for concerted DNA integration *in vitro*. J Mol Biol. (2024) 436:168557. doi: 10.1016/j.jmb.2024.168557. PMID: 38582148 PMC11134455

[27] Li M Li Z Chen X Cui Y Engelman A Craigie R . HIV-1 intasomes assembled with excess integrase C-terminal domain protein facilitate structural studies by cryo-EM and reveal the role of the integrase C-terminal tail in HIV-1 integration. Viruses. (2024) 16(7):1166. doi: 10.3390/v16071166. PMID: 39066328 PMC11281638

[28] Davids B Balasubramaniam M Sapp N Prakash P Ingram S Li M . Human three prime repair exonuclease 1 promotes HIV-1 integration by preferentially degrading unprocessed viral DNA. J Virol. (2021) 95:e0055521. doi: 10.1128/jvi.00555-21. PMID: 34105995 PMC8354242

[29] Jóźwik I Li W Zhang D Wong D Grawenhoff J Ballandras-Colas A . B-to-A transition in target DNA during retroviral integration. Nucleic Acids Res. (2022) 50:8898–918. doi: 10.1093/nar/gkac644. PMID: 35947647 PMC9410886

[30] DiScipio K Weerasooriya S Szczepaniak R Hazeen A Wright L Wright D . Two-metal ion-dependent enzymes as potential antiviral targets in human herpesviruses. mBio. (2022) 13:e0322621. doi: 10.1128/mbio.03226-21. PMID: 35073739 PMC8787488

[31] Dam K Fan C Yang Z Bjorkman P . Intermediate conformations of CD4-bound HIV-1 Env heterotrimers. Nature. (2023) 623:1017–25. doi: 10.1038/s41586-023-06639-8. PMID: 37993719 PMC10686819

[32] Nguyen H Wang Q Anang S Sodroski J . Characterization of the human immunodeficiency virus (HIV-1) envelope glycoprotein conformational states on infectious virus particles. J Virol. (2023) 97:e0185722. doi: 10.1128/jvi.01857-22. PMID: 36815832 PMC10062176

[33] Murakami T Ono A . HIV-1 entry: Duels between Env and host antiviral transmembrane proteins on the surface of virus particles. Curr Opin Virol. (2021) 50:59–68. doi: 10.1016/j.coviro.2021.07.005. PMID: 34390925 PMC8500929

[34] Chiliveri S Louis J Best R Bax A . Real-time exchange of the lipid-bound intermediate and post-fusion states of the HIV-1 gp41 ectodomain. J Mol Biol. (2022) 434:167683. doi: 10.1016/j.jmb.2022.167683. PMID: 35700771 PMC9378563

[35] Sarkar S Zadrozny K Zadorozhnyi R Russell R Quinn C Kleinpeter A . Structural basis of HIV-1 maturation inhibitor binding and activity. Nat Commun. (2023) 14:1237. doi: 10.1038/s41467-023-36569-y. PMID: 36871077 PMC9985623

[36] Morales-Ramirez J Bogner J Molina J Lombaard J Dicker I Stock D . Safety, efficacy, and dose response of the maturation inhibitor GSK3532795 (formerly known as BMS-955176) plus tenofovir/emtricitabine once daily in treatment-naive HIV-1-infected adults: Week 24 primary analysis from a randomized Phase IIb trial. PloS One. (2018) 13:e0205368. doi: 10.1371/journal.pone.0205368. PMID: 30352054 PMC6198970

[37] Dick A Cocklin S . Recent advances in HIV-1 Gag inhibitor design and development. Molecules. (2020) 25(7):1687. doi: 10.3390/molecules25071687. PMID: 32272714 PMC7181048

[38] Kozal M Aberg J Pialoux G Cahn P Thompson M Molina J . Fostemsavir in adults with multidrug-resistant HIV-1 infection. N Engl J Med. (2020) 382:1232–43. doi: 10.1056/NEJMoa1902493. PMID: 32212519

[39] Hitchcock A Kufel W Dwyer K Sidman E . Lenacapavir: A novel injectable HIV-1 capsid inhibitor. Int J Antimicrob Agents. (2024) 63:107009. doi: 10.1016/j.ijantimicag.2023.107009. PMID: 37844807

[40] Cilento M Kirby K Sarafianos S . Avoiding drug resistance in HIV reverse transcriptase. Chem Rev. (2021) 121:3271–96. doi: 10.1021/acs.chemrev.0c00967. PMID: 33507067 PMC8149104

[41] Fernandes L Lopes J Bonjorno A Prates J Scarim C Dos Santos J . The application of prodrugs as a tool to enhance the properties of nucleoside reverse transcriptase inhibitors. Viruses. (2023) 15(11):2234. doi: 10.3390/v15112234. PMID: 38005911 PMC10675571

[42] Mimtsoudis I Tsachouridou O Akinosoglou K Metallidis S . Treatment management challenges in naïve and experienced HIV-1-infected individuals carrying the M184V mutation. Viruses. (2024) 16(9):1392. doi: 10.3390/v16091392. PMID: 39339868 PMC11437411

[43] Boopathy A D’Antoni M Andreatta K Chang S Hindman J Callebaut C . Brief report: Efficacy of bictegravir/emtricitabine/tenofovir alafenamide in participants with preexisting K65R/N in HIV-1 in Phase 2/3/3b clinical trials. J Acquir Immune Defic Syndr. (2025) 100:336–41. doi: 10.1097/qai.0000000000003742. PMID: 40779404

[44] Byun H Papathanasopoulos M Steegen K Basson A . Thymidine analogue mutations with M184V significantly decrease phenotypic susceptibility of HIV-1 subtype C reverse transcriptase to islatravir. Viruses. (2024) 16(12):1888. doi: 10.3390/v16121888. PMID: 39772195 PMC11680407

[45] Wang Z Rumrill S Kang D Guma S Feng D De Clercq E . Development of enhanced HIV-1 non-nucleoside reverse transcriptase inhibitors with improved resistance and pharmacokinetic profiles. Sci Adv. (2025) 11:eadt8916. doi: 10.1126/sciadv.adt8916. PMID: 40446037 PMC12124364

[46] Ha B Larsen K Zhang J Fu Z Montabana E Jackson L . High-resolution view of HIV-1 reverse transcriptase initiation complexes and inhibition by NNRTI drugs. Nat Commun. (2021) 12:2500. doi: 10.1038/s41467-021-22628-9. PMID: 33947853 PMC8096811

[47] Cecchini D Cassetti I Scarnato F Fiori A Nuevo J Villaverde C . Prevalence of Doravirine resistance mutations in a large-scale HIV-1 transmitted drug resistance survey in Buenos Aires, Argentina. Viruses. (2025) 17(5):731. doi: 10.3390/v17050731. PMID: 40431742 PMC12116006

[48] Brenner B Oliveira M Ibanescu R Routy J Thomas R . Doravirine responses to HIV-1 viruses bearing mutations to NRTIs and NNRTIs under *in vitro* selective drug pressure. J Antimicrob Chemother. (2023) 78:1921–8. doi: 10.1093/jac/dkad184. PMID: 37303226

[49] Janssens J De Wit F Parveen N Debyser Z . Single-cell imaging shows that the transcriptional state of the HIV-1 provirus and its reactivation potential depend on the integration site. mBio. (2022) 13:e0000722. doi: 10.1128/mbio.00007-22. PMID: 35708287 PMC9426465

[50] Jing T Shan Z Dinh T Biswas A Jang S Greenwood J . Oligomeric HIV-1 integrase structures reveal functional plasticity for intasome assembly and RNA binding. Nat Commun. (2025) 16:9430. doi: 10.1038/s41467-025-64479-8. PMID: 41136407 PMC12552619

[51] Koutná E Lux V Kouba T Škerlová J Nováček J Srb P . Multivalency of nucleosome recognition by LEDGF. Nucleic Acids Res. (2023) 51:10011–25. doi: 10.1093/nar/gkad674. PMID: 37615563 PMC10570030

[52] Singh P Li W Bedwell G Fadel H Poeschla E Engelman AN . Allosteric integrase inhibitor influences on HIV-1 integration and roles of LEDGF/p75 and HDGFL2 host factors. Viruses. (2022) 14(9):1883. doi: 10.3390/v14091883. PMID: 36146690 PMC9502684

[53] Ferrer P Ramos V Durán M Maureira D Beltrán C Afani A . Prevalence of acquired resistance to HIV integrase strand transfer inhibitors (INSTIs) in clinical samples from treatment-experienced patients in Chile, 2012-2023. J Virus Erad. (2025) 11:100608. doi: 10.1016/j.jve.2025.100608. PMID: 41019769 PMC12476102

[54] Buzon-Martin L Navarro-San Francisco C Fernandez-Regueras M Sanchez-Gomez L . Integrase strand transfer inhibitor resistance mediated by R263K plus E157Q in a patient with HIV infection treated with bictegravir/tenofovir alafenamide/emtricitabine: case report and review of the literature. J Antimicrob Chemother. (2024) 79:1153–6. doi: 10.1093/jac/dkae085. PMID: 38558010

[55] Jette C Barnes C Kirk S Melillo B Smith A Bjorkman P . Cryo-EM structures of HIV-1 trimer bound to CD4-mimetics BNM-III-170 and M48U1 adopt a CD4-bound open conformation. Nat Commun. (2021) 12:1950. doi: 10.1038/s41467-021-21816-x. PMID: 33782388 PMC8007822

[56] Blair H . Ibalizumab: a review in multidrug-resistant HIV-1 infection. Drugs. (2020) 80:189–96. doi: 10.1007/s40265-020-01258-3. PMID: 31970712

[57] Le Hingrat Q Collin G Bachelard A Ghosn J Chalal S Pacanowski J . Ibalizumab shows in-vitro activity against group A and group B HIV-2 clinical isolates. Aids. (2022) 36:1055–60. doi: 10.1097/qad.0000000000003218. PMID: 35262531

[58] Chatterjee D Niu L Medjahed H Ding S Benlarbi M Bélanger É . A gp41 HR2 residue modulates the susceptibility of HIV-1 envelope glycoproteins to small molecule inhibitors targeting gp120. J Virol. (2025) 99:e0226724. doi: 10.1128/jvi.02267-24. PMID: 40145736 PMC11998491

[59] Wang Q Finzi A Sodroski J . The conformational states of the HIV-1 envelope glycoproteins. Trends Microbiol. (2020) 28:655–67. doi: 10.1016/j.tim.2020.03.007. PMID: 32418859 PMC7363548

[60] Bird G Patten J Zavadoski W Barucci N Godes M Moyer B . A stapled lipopeptide platform for preventing and treating highly pathogenic viruses of pandemic potential. Nat Commun. (2024) 15:274. doi: 10.1038/s41467-023-44361-1. PMID: 38177138 PMC10766962

[61] Beran C Duggan J Sahloff E . A narrative review of novel agents for managing heavily treatment-experienced people living with HIV. J Pharm Technol. (2024) 40:194–201. doi: 10.1177/87551225241259894. PMID: 39157636 PMC11325682

[62] Su B Yao C Zhao Q Cai W Wang M Lu H . Efficacy and safety of the long-acting fusion inhibitor albuvirtide in antiretroviral-experienced adults with human immunodeficiency virus-1: interim analysis of the randomized, controlled, phase 3, non-inferiority TALENT study. Chin Med J (Engl). (2020) 133:2919–27. doi: 10.1097/cm9.0000000000001273. PMID: 33252379 PMC7752691

[63] Mohamed H Gurrola T Berman R Collins M Sariyer I Nonnemacher M . Targeting CCR5 as a component of an HIV-1 therapeutic strategy. Front Immunol. (2021) 12:816515. doi: 10.3389/fimmu.2021.816515. PMID: 35126374 PMC8811197

[64] Moore K Thakkar N Magee M Sevinsky H Vakkalagadda B Lubin S . Pharmacokinetics of temsavir, the active moiety of the HIV-1 attachment inhibitor prodrug, fostemsavir, coadministered with cobicistat, etravirine, darunavir/cobicistat, or darunavir/ritonavir with or without etravirine in healthy participants. Antimicrob Agents Chemother. (2022) 66:e0225121. doi: 10.1128/aac.02251-21. PMID: 35315687 PMC9017385

[65] Prévost J Chen Y Zhou F Tolbert W Gasser R Medjahed H . Structure-function analyses reveal key molecular determinants of HIV-1 CRF01_AE resistance to the entry inhibitor temsavir. Nat Commun. (2023) 14:6710. doi: 10.1038/s41467-023-42500-2. PMID: 37872202 PMC10593844

[66] Richard J Prévost J Bourassa C Brassard N Boutin M Benlarbi M . Temsavir blocks the immunomodulatory activities of HIV-1 soluble gp120. Cell Chem Biol. (2023) 30:540–52.e6. doi: 10.1016/j.chembiol.2023.03.003. PMID: 36958337 PMC10198848

[67] Monteiro A Yu K Hicar M . Peptide-based fusion inhibitors for preventing the six-helix bundle formation of class I fusion proteins: HIV and beyond. Curr HIV Res. (2021) 19:465–75. doi: 10.2174/1570162x19666210908115231. PMID: 34503415 PMC9166635

[68] Schiffner T Phung I Ray R Irimia A Tian M Swanson O . Vaccination induces broadly neutralizing antibody precursors to HIV gp41. Nat Immunol. (2024) 25:1073–82. doi: 10.1038/s41590-024-01833-w. PMID: 38816615 PMC11147780

[69] Xiao T Frey G Fu Q Lavine C Scott D Seaman M . HIV-1 fusion inhibitors targeting the membrane-proximal external region of Env spikes. Nat Chem Biol. (2020) 16:529–37. doi: 10.1038/s41589-020-0496-y. PMID: 32152540 PMC7723321

[70] Stelzle D Rangaraj A Jarvis J Razakasoa N Perrin G Low-Beer D . Prevalence of advanced HIV disease in sub-Saharan Africa: a multi-country analysis of nationally representative household surveys. Lancet Glob Health. (2025) 13:e437–e46. doi: 10.1016/s2214-109x(24)00538-2. PMID: 40021302 PMC11868778

[71] Gandhi R Landovitz R Sax P Smith D Springer S Günthard H . Antiretroviral drugs for treatment and prevention of HIV in adults: 2024 recommendations of the International Antiviral Society-USA Panel. Jama. (2025) 333:609–28. doi: 10.1001/jama.2024.24543. PMID: 39616604

[72] Moyle G Assoumou L de Castro N Post F Curran A Rusconi S . Switching to dolutegravir plus rilpivirine versus maintaining current antiretroviral therapy regimen in virologically suppressed people with HIV-1 and the Lys103Asn (K103N) mutation: 48-week results from a randomised, open-label pilot clinical trial. Lancet HIV. (2024) 11:e156–e66. doi: 10.1016/s2352-3018(23)00292-8. PMID: 38417976

[73] Jaeger H Overton E Richmond G Rizzardini G Andrade-Villanueva J Mngqibisa R . Long-acting cabotegravir and rilpivirine dosed every 2 months in adults with HIV-1 infection (ATLAS-2M), 96-week results: a randomised, multicentre, open-label, phase 3b, non-inferiority study. Lancet HIV. (2021) 8:e679–e89. doi: 10.1016/s2352-3018(21)00185-5. PMID: 34648734

[74] Overton E Richmond G Rizzardini G Thalme A Girard P Wong A . Long-acting cabotegravir and rilpivirine dosed every 2 months in adults with human immunodeficiency virus 1 type 1 infection: 152-week results from ATLAS-2M, a randomized, open-label, phase 3b, noninferiority study. Clin Infect Dis. (2023) 76:1646–54. doi: 10.1093/cid/ciad020. PMID: 36660819 PMC10156123

[75] Segal-Maurer S DeJesus E Stellbrink H Castagna A Richmond G Sinclair G . Capsid inhibition with lenacapavir in multidrug-resistant HIV-1 infection. N Engl J Med. (2022) 386:1793–803. doi: 10.1056/NEJMoa2115542. PMID: 35544387

[76] Ogbuagu O Molina J Chetchotisakd P Ramgopal M Sanchez W Brunetta J . Efficacy and safety of long-acting subcutaneous lenacapavir in heavily treatment-experienced people with multidrug-resistant HIV-1: week 104 results of a phase 2/3 trial. Clin Infect Dis. (2025) 80:566–74. doi: 10.1093/cid/ciae423. PMID: 39206943

[77] Eron J Little S Crofoot G Cook P Ruane P Jayaweera D . Safety of teropavimab and zinlirvimab with lenacapavir once every 6 months for HIV treatment: a phase 1b, randomised, proof-of-concept study. Lancet HIV. (2024) 11:e146–e55. doi: 10.1016/s2352-3018(23)00293-x. PMID: 38307098 PMC12245405

[78] Kantor R Pau A Kozal M Hyle E . Do we need routine integrase resistance testing before starting antiretroviral therapy? Lancet HIV. (2025) 12:e664–e8. doi: 10.1016/s2352-3018(25)00108-0. PMID: 40541216 PMC12265005

[79] Nutt C Lessells R Muyindike W Johnson L Kouyos R Loosli T . Approaches to the management of virologic failure on dolutegravir-based antiretroviral therapy in the 50 countries with the highest adult HIV prevalence. Clin Infect Dis. (2026) 82:265–73. doi: 10.1093/cid/ciaf444. PMID: 40795893 PMC12380185

[80] Zaçe D Rindi L Compagno M Colagrossi L Santoro M Andreoni M . Managing low-level HIV viraemia in antiretroviral therapy: a systematic review and meta-analysis. Sex Transm Infect. (2024) 100:460–8. doi: 10.1136/sextrans-2024-056198. PMID: 39288983 PMC11503136

[81] Esteban-Cantos A Montejano R Pinto-Martínez A Rodríguez-Centeno J Pulido F Arribas J . Non-suppressible viraemia during HIV-1 therapy: a challenge for clinicians. Lancet HIV. (2024) 11:e333–e40. doi: 10.1016/s2352-3018(24)00063-8. PMID: 38604202

[82] Campagna R Nonne C Antonelli G Turriziani O . Archived HIV-1 drug resistance mutations: role of proviral HIV-1 DNA genotype for the management of virological responder people living with HIV. Viruses. (2024) 16(11):1697. doi: 10.3390/v16111697. PMID: 39599811 PMC11599110

[83] Andreatta K Sax P Wohl D D’Antoni M Liu H Hindman J . Efficacy of bictegravir/emtricitabine/tenofovir alafenamide versus dolutegravir-based three-drug regimens in people with HIV with varying adherence to antiretroviral therapy. J Antimicrob Chemother. (2025) 80:281–91. doi: 10.1093/jac/dkae407. PMID: 39556192 PMC11695908

[84] Esser S Brunetta J Inciarte A Levy I D’Arminio Monforte A Lambert J . Twelve-month effectiveness and safety of bictegravir/emtricitabine/tenofovir alafenamide in people with HIV: real-world insights from BICSTaR cohorts. HIV Med. (2024) 25:440–53. doi: 10.1111/hiv.13593. PMID: 38148567

[85] Bekker L Das M Abdool Karim Q Ahmed K Batting J Brumskine W . Twice-yearly lenacapavir or daily F/TAF for HIV prevention in cisgender women. N Engl J Med. (2024) 391:1179–92. doi: 10.1056/NEJMoa2407001. PMID: 39046157

[86] Armani-Tourret M Bone B Tan T Sun W Bellefroid M Struyve T . Immune targeting of HIV-1 reservoir cells: a path to elimination strategies and cure. Nat Rev Microbiol. (2024) 22:328–44. doi: 10.1038/s41579-024-01010-8. PMID: 38337034 PMC11131351

[87] Kinloch N Shahid A Dong W Kirkby D Jones B Beelen C . HIV reservoirs are dominated by genetically younger and clonally enriched proviruses. mBio. (2023) 14:e0241723. doi: 10.1128/mbio.02417-23. PMID: 37971267 PMC10746175

[88] Teixeira A Bittar C Silva Santos G Oliveira T Huang A Linden N . Transcription of HIV-1 at sites of intact latent provirus integration. Preprint. bioRxiv. (2024) 2024.04.26.591331. doi: 10.1101/2024.04.26.591331. PMID: 39141127 PMC11323366

[89] Vellas C Nayrac M Collercandy N Requena M Jeanne N Latour J . Intact proviruses are enriched in the colon and associated with PD-1(+)TIGIT(-) mucosal CD4(+) T cells of people with HIV-1 on antiretroviral therapy. EBioMedicine. (2024) 100:104954. doi: 10.1016/j.ebiom.2023.104954. PMID: 38160480 PMC10792747

[90] Peterson J Chandel S James K Bennett E Wu V White C . Single-cell characterization of the gastrointestinal HIV reservoir reveals heterogeneous cellular phenotypes. J Clin Invest. (2026) 136(4):e196536. doi: 10.1172/jci196536. PMID: 41433124 PMC12904708

[91] Gallego-Cortés A Sánchez-Gaona N Mancebo-Pérez C Ruiz-Isant O Benítez-Martínez A Landolfi S . Identification of inducible HIV reservoirs in tonsillar, intestinal and cervical tissue models of HIV latency. Nat Commun. (2025) 16:10353. doi: 10.1038/s41467-025-65288-9. PMID: 41285793 PMC12645015

[92] Ling L Kim M Soper A Kovarova M Spagnuolo R Begum N . Analysis of the effect of HDAC inhibitors on the formation of the HIV reservoir. mBio. (2024) 15:e0163224. doi: 10.1128/mbio.01632-24. PMID: 39136440 PMC11389399

[93] Irrinki A Kaur J Randhawa B McFadden R Snyder C Truong H . Activating PKC-ϵ induces HIV expression with improved tolerability. PloS Pathog. (2025) 21:e1012874. doi: 10.1371/journal.ppat.1012874. PMID: 39913544 PMC11801715

[94] Tioka L Diez R Sönnerborg A van de Klundert M . Latency reversing agents and the road to an HIV cure. Pathogens. (2025) 14(3):232. doi: 10.3390/pathogens14030232. PMID: 40137717 PMC11944434

[95] Matsuda K Maeda K . HIV reservoirs and treatment strategies toward curing HIV infection. Int J Mol Sci. (2024) 25(5):2621. doi: 10.3390/ijms25052621. PMID: 38473868 PMC10932120

[96] Deeks S Archin N Cannon P Collins S Jones R de Jong M . Research priorities for an HIV cure: International AIDS Society Global Scientific Strategy 2021. Nat Med. (2021) 27:2085–98. doi: 10.1038/s41591-021-01590-5. PMID: 34848888

[97] Margolis D Archin N Cohen M Eron J Ferrari G Garcia J . Curing HIV: seeking to target and clear persistent infection. Cell. (2020) 181:189–206. doi: 10.1016/j.cell.2020.03.005. PMID: 32220311 PMC7896558

[98] Mori L Corley M McAuley A Pang A Venables T Ndhlovu L . Transcriptional and methylation outcomes of didehydro-cortistatin A use in HIV-1-infected CD4+ T cells. Life Sci Alliance. (2024) 7(10):e202402653. doi: 10.26508/lsa.202402653. PMID: 39089880 PMC11294679

[99] Pellaers E Denis A Debyser Z . New latency-promoting agents for a block-and-lock functional cure strategy. Curr Opin HIV AIDS. (2024) 19:95–101. doi: 10.1097/coh.0000000000000844. PMID: 38457209 PMC10990034

[100] Claiborne D Detwiler Z Docken S Borland T Cromer D Simkhovich A . High frequency CCR5 editing in human hematopoietic stem progenitor cells protects xenograft mice from HIV infection. Nat Commun. (2025) 16:446. doi: 10.1038/s41467-025-55873-3. PMID: 39774003 PMC11707138

[101] Lewin SR Bansbach C Kemps D Mathae L Das KT McCune JM . Target product profile for cell-based and gene-based therapies to achieve a cure for HIV. Lancet HIV. (2025) 12:e154–62. doi: 10.1016/s2352-3018(24)00277-7. PMID: 39761679

[102] Dudek AM Feist WN Sasu EJ Luna SE Ben-Efraim K Bak RO . A simultaneous knockout knockin genome editing strategy in HSPCs potently inhibits CCR5- and CXCR4-tropic HIV-1 infection. Cell Stem Cell. (2024) 31:499–518.e6. doi: 10.1016/j.stem.2024.03.002. PMID: 38579682 PMC11212398

[103] Jensen BO Knops E Cords L Lübke N Salgado M Busman-Sahay K . In-depth virological and immunological characterization of HIV-1 cure after CCR5Δ32/Δ32 allogeneic hematopoietic stem cell transplantation. Nat Med. (2023) 29:583–7. doi: 10.1038/s41591-023-02213-x. PMID: 36807684 PMC10033413

[104] Gaebler C Kor S Allers K Perotti M Mwangi D Meixenberger K . Sustained HIV-1 remission after heterozygous CCR5Δ32 stem cell transplantation. Nature. (2026) 650(8102):701–9. doi: 10.1038/s41586-025-09893-0. PMID: 41326734 PMC12916306

[105] Sáez-Cirión A Mamez AC Avettand-Fenoel V Nabergoj M Passaes C Thoueille P . Sustained HIV remission after allogeneic hematopoietic stem cell transplantation with wild-type CCR5 donor cells. Nat Med. (2024) 30:3544–54. doi: 10.1038/s41591-024-03277-z. PMID: 39222660 PMC11645271

[106] Liu B Zhang W Xia B Jing S Du Y Zou F . Broadly neutralizing antibody-derived CAR T cells reduce viral reservoir in individuals infected with HIV-1. J Clin Invest. (2021) 131(19):e150211. doi: 10.1172/jci150211. PMID: 34375315 PMC8483761

[107] Anthony-Gonda K Ray A Su H Wang Y Xiong Y Lee D . *In vivo* killing of primary HIV-infected cells by peripheral-injected early memory-enriched anti-HIV duoCAR T cells. JCI Insight. (2022) 7(21):e161698. doi: 10.1172/jci.insight.161698. PMID: 36345941 PMC9675454

[108] Mao Y Liao Q Zhu Y Bi M Zou J Zheng N . Efficacy and safety of novel multifunctional M10 CAR-T cells in HIV-1-infected patients: a phase I, multicenter, single-arm, open-label study. Cell Discov. (2024) 10:49. doi: 10.1038/s41421-024-00658-z. PMID: 38740803 PMC11091177

[109] Pan H Yang X Wang J Liang H Jiang Z Zhao L . Allogeneic gene-edited HIV-specific CAR-T cells secreting PD-1 blocking scFv enhance specific cytotoxic activity against HIV Env(+) cells invivo. Virol Sin. (2023) 38:285–95. doi: 10.1016/j.virs.2023.01.003. PMID: 36657565 PMC10176442

[110] Mancuso P Chen C Kaminski R Gordon J Liao S Robinson JA . CRISPR based editing of SIV proviral DNA in ART treated non-human primates. Nat Commun. (2020) 11:6065. doi: 10.1038/s41467-020-19821-7. PMID: 33247091 PMC7695718

[111] Dash PK Chen C Kaminski R Su H Mancuso P Sillman B . CRISPR editing of CCR5 and HIV-1 facilitates viral elimination in antiretroviral drug-suppressed virus-infected humanized mice. Proc Natl Acad Sci USA. (2023) 120:e2217887120. doi: 10.1073/pnas.2217887120. PMID: 37126704 PMC10175831

[112] Fan M Berkhout B Herrera-Carrillo E . A combinatorial CRISPR-Cas12a attack on HIV DNA. Mol Ther Methods Clin Dev. (2022) 25:43–51. doi: 10.1016/j.omtm.2022.02.010. PMID: 35356755 PMC8933334

[113] Herskovitz J Hasan M Patel M Blomberg WR Cohen JD Machhi J . CRISPR-cas9 mediated exonic disruption for HIV-1 elimination. EBioMedicine. (2021) 73:103678. doi: 10.1016/j.ebiom.2021.103678. PMID: 34774454 PMC8633974

[114] Ma Y Wei W Yang Z Zhou Y Dong T Wang T . Exosomes as nonviral carrier for targeted delivery of CRISPR-Cas12a for therapeutic HIV-1 proviral DNA editing. Mol Ther. (2026) 34:1152–71. doi: 10.1016/j.ymthe.2025.11.012. PMID: 41229123 PMC12882320

[115] Wu C Johnson NM Yu S Lo AS Sahu GK Marx PA . Persistence of CMV-specific anti-HIV CAR T cells after adoptive immunotherapy. J Virol. (2025) 99:e0193324. doi: 10.1128/jvi.01933-24. PMID: 40207929 PMC12090794

[116] Guan M Lim L Holguin L Han T Vyas V Urak R . Pre-clinical data supporting immunotherapy for HIV using CMV-HIV-specific CAR T cells with CMV vaccine. Mol Ther Methods Clin Dev. (2022) 25:344–59. doi: 10.1016/j.omtm.2022.04.007. PMID: 35573050 PMC9062763

[117] Su H Mueller A Goldstein H . Recent advances on anti-HIV chimeric antigen receptor-T-cell treatment to provide sustained HIV remission. Curr Opin HIV AIDS. (2024) 19:169–78. doi: 10.1097/coh.0000000000000858. PMID: 38695148 PMC11981014

[118] Cevaal PM Kan S Fisher BM Moso MA Tan A Liu H . Efficient mRNA delivery to resting T cells to reverse HIV latency. Nat Commun. (2025) 16:4979. doi: 10.1038/s41467-025-60001-2. PMID: 40442114 PMC12122926

[119] Rust BJ Kean LS Colonna L Brandenstein KE Poole NH Obenza W . Robust expansion of HIV CAR T cells following antigen boosting in ART-suppressed nonhuman primates. Blood. (2020) 136:1722–34. doi: 10.1182/blood.2020006372. PMID: 32614969 PMC7544543

[120] Lanz C Meier J Stöckle M Furrer H Calmy A Cavassini M . HIV-1 low-level viremia predicts viral failure in participants on antiretroviral therapy in the swiss HIV cohort study. Clin Infect Dis. (2025) 81:57–66. doi: 10.1093/cid/ciae569. PMID: 39570670 PMC12314502

[121] Weerasuria M McMahon JH Lewin SR Lau JSY . The role of analytical treatment interruptions in shaping HIV-specific immunity and HIV cure. Curr Opin HIV AIDS. (2025) 20:543–51. doi: 10.1097/coh.0000000000000973. PMID: 40833597 PMC12517717

[122] Gianella S Yu T Wang R Ignacio C Schanz M Kouyos RD . Viral and immune risk factors of HIV rebound after interruption of antiretroviral therapy. J Infect Dis. (2025) 231:1221–9. doi: 10.1093/infdis/jiae585. PMID: 39661441 PMC12128076

[123] McCluskey SM Gandhi M . Predictors of treatment-emergent resistance to dolutegravir. Lancet HIV. (2025) 12:e660–3. doi: 10.1016/s2352-3018(25)00127-4. PMID: 40544855 PMC12232622

[124] D2EFT Study Group . Dolutegravir plus boosted darunavir versus recommended standard-of-care antiretroviral regimens in people with HIV-1 for whom recommended first-line non-nucleoside reverse transcriptase inhibitor therapy has failed (D2EFT): an open-label, randomised, phase 3b/4 trial. Lancet HIV. (2024) 11(7):e436–48. doi: 10.1016/s2352-3018(24)00089-4. PMID: 38788744 PMC11700562

[125] Kelley CF Acevedo-Quiñones M Agwu AL Avihingsanon A Benson P Blumenthal J . Twice-yearly lenacapavir for HIV prevention in men and gender-diverse persons. N Engl J Med. (2025) 392:1261–76. doi: 10.1056/NEJMoa2411858. PMID: 39602624

[126] Colasanti JA Aldredge A Niles-Carnes LV Ochieng E Pareek P Robinson V . Long-acting cabotegravir/rilpivirine, lenacapavir, and ibalizumab use among persons with HIV-1 viremia at a Ryan White-funded clinic in the urban U.S. South. Clin Infect Dis. (2026) 82(4):564–74. doi: 10.1093/cid/ciaf425. PMID: 40747944 PMC13131956

[127] Bachelard A Le Hingrat Q Ferré VM Lê M Peytavin G Damond F . Salvage therapy including foscarnet and ibalizumab for multidrug-resistant human immunodeficiency virus type 2 infection. Clin Infect Dis. (2024) 78:1005–10. doi: 10.1093/cid/ciad695. PMID: 38630945

[128] Pereira M Vale N . Saquinavir: from HIV to COVID-19 and cancer treatment. Biomolecules. (2022) 12(7):944. doi: 10.3390/biom12070944. PMID: 35883499 PMC9313067

[129] Joshi SR Masiá M Mitha E Castagna A Cordova E Ramgopal M . Efficacy and safety of the HIV-1 maturation inhibitor GSK3640254 plus dolutegravir as a two-drug regimen in adults naive to antiretroviral therapy (DYNAMIC): 24-week results from a randomised phase 2b study. EClinicalMedicine. (2025) 89:103566. doi: 10.1016/j.eclinm.2025.103566. PMID: 41357331 PMC12675015

[130] DeJesus E Harward S Jewell RC Johnson M Dumont E Wilches V . A phase IIa study evaluating safety, pharmacokinetics, and antiviral activity of GSK2838232, a novel, second-generation maturation inhibitor, in participants with human immunodeficiency virus type 1 infection. Clin Infect Dis. (2020) 71:1255–62. doi: 10.1093/cid/ciz938. PMID: 31769793

[131] Patel RR Hoover KW Lale A Cabrales J Byrd KM Kourtis AP . Clinical recommendation for the use of injectable lenacapavir as HIV preexposure prophylaxis - United States, 2025. MMWR Morb Mortal Wkly Rep. (2025) 74:541–9. doi: 10.15585/mmwr.mm7435a1. PMID: 40966169 PMC12445877

[132] Colson AE Crofoot GE Ruane PJ Ramgopal MN Dretler AW Nahass RG . Once-weekly oral islatravir plus lenacapavir versus daily oral bictegravir, emtricitabine, and tenofovir alafenamide in persons with HIV-1: A phase 2 randomized study. Ann Intern Med. (2026) 179(2):168–76. doi: 10.7326/annals-25-01939. PMID: 41429026

[133] Edupuganti S Hurt CB Stephenson KE Huang Y Paez CA Yu C . Safety, tolerability, pharmacokinetics, and neutralisation activities of the anti-HIV-1 monoclonal antibody PGT121.414.LS administered alone and in combination with VRC07-523LS in adults without HIV in the USA (HVTN 136/HPTN 092): a first-in-human, open-label, randomised controlled phase 1 trial. Lancet HIV. (2025) 12:e13–25. doi: 10.1016/s2352-3018(24)00247-9. PMID: 39667379 PMC11795396

[134] Seaton KE Paez CA Yu C Karuna ST Gamble T Miner MD . Safety, pharmacokinetics, and neutralisation activity of PGDM1400LS, a V2 specific HIV-1 broadly neutralising antibody, infused intravenously or subcutaneously in people without HIV-1 in the USA (HVTN 140/HPTN 101 part A): a first-in-human, phase 1 randomised trial. Lancet HIV. (2025) 12:e405–15. doi: 10.1016/s2352-3018(25)00012-8. PMID: 40441807 PMC12266790

[135] Awan SF Pegu A Strom L Carter CA Hendel CS Holman LA . Phase 1 trial evaluating safety and pharmacokinetics of HIV-1 broadly neutralizing mAbs 10E8VLS and VRC07-523LS. JCI Insight. (2024) 9(7):e175375. doi: 10.1172/jci.insight.175375. PMID: 38587079 PMC11128198

[136] Cunningham CK McFarland EJ Muresan P Capparelli EV Perlowski C Johnston B . Safety, tolerability, and pharmacokinetics of long-acting broadly neutralizing HIV-1 monoclonal antibody VRC07-523LS in newborn infants exposed to HIV-1. J Pediatr Infect Dis Soc. (2025) 14(2):piaf002. doi: 10.1093/jpids/piaf002. PMID: 39821046 PMC11799610

[137] Wu RL Houser KV Gaudinski MR Widge AT Awan SF Carter CA . Safety and pharmacokinetics of N6LS, a broadly neutralising monoclonal antibody for HIV: a phase 1, open-label, dose-escalation study in healthy adults. Lancet HIV. (2025) 12:e485–95. doi: 10.1016/s2352-3018(25)00041-4. PMID: 40409326 PMC12282510

[138] Priddy FH Lewis DJM Gelderblom HC Hassanin H Streatfield C LaBranche C . Adeno-associated virus vectored immunoprophylaxis to prevent HIV in healthy adults: a phase 1 randomised controlled trial. Lancet HIV. (2019) 6:e230–9. doi: 10.1016/s2352-3018(19)30003-7. PMID: 30885692 PMC6443625

